# *In vitro* skin models to study epithelial regeneration from the hair follicle

**DOI:** 10.1371/journal.pone.0174389

**Published:** 2017-03-28

**Authors:** Nkemcho Ojeh, Baki Akgül, Marjana Tomic-Canic, Mike Philpott, Harshad Navsaria

**Affiliations:** 1 Centre for Cutaneous Research, Blizard Institute, Bart’s & The London School of Medicine and Dentistry, London, United Kingdom; 2 Institute of Virology, University of Cologne, Cologne, Germany; 3 Department of Dermatology and Cutaneous Surgery, University of Miami, Miller School of Medicine, Miami, Florida, United States of America; University of Alabama at Birmingham, UNITED STATES

## Abstract

The development of dermal equivalents (DEs) for the treatment of burns has contributed toward efficient wound closure. A collagen-glycosaminoglycan DE (C-GAG) grafted with hair follicles converted a full-thickness wound to partial-thickness resulting in complete wound closure and restored hair. In this study we compared the ability of both intact pilosebaceous units (PSU) or truncated hair follicles (THF) to regenerate a multilayered epidermis *in vitro* when implanted into de-epidermalized dermis (DED) or C-GAG with the epidermis generated *in vivo* using C-CAG. Keratinocytes explanted from the outer root sheath of PSU and THF in both DED and C-GAG but only formed a multilayered epidermis with PSU in DED. PSU were more effective at forming multilayered epidermis in DED than THF. Both DED and C-GAG skin expressed proliferation (PCNA), differentiation (K1, K10), hyperproliferation (K6, K16), basal (K14), putative stem cell (p63), extracellular matrix protein (Collagen IV), mesenchymal (vimentin) and adherens junction (β-catenin) markers. These data suggest DEs supported initial maintenance of the implanted hair follicles, in particular PSU, and provide an excellent model with which to investigate the regulation of hair follicle progenitor epithelial cells during epidermal regeneration. Although neither PSU nor THF formed multilayered epidermis in C-CAG *in vitro*, hair follicles implanted into engrafted C-GAG on a burns patient resulted in epithelial regeneration and expression of proliferation and differentiation markers in a similar manner to that seen *in vitro*. However, the failure of C-GAG to support epidermal regeneration *in vitro* suggests *in vivo* factors are essential for full epidermal regeneration using C-GAG.

## Introduction

Early burn wounds excision followed by wound closure is the key to success for the treatment of large burn injuries and subsequent increase in survival rates [1]. Split-thickness and allogenic skin grafts are typically used for the resurfacing of burn wounds. The former provides permanent cover but, availability can be restricted due to sparse donor sites which can lead to pain and scar formation. The latter are temporary, subject to rejection, and pose a risk of viral transmission. Utilisation of keratinocyte sheets in the treatment of burns has also been documented [2]. However, due to the flat dermo-epidermal junction, these sheets are fragile on full-thickness wounds and are prone to blistering thus influencing take rates [3]. Poor take rates can be improved by grafting these sheets in combination with a dermal component [4].

Dermal equivalents (DEs) can be cellular or acellular and can include biodegradable or nonbiodegradable polymers. Acellular DE, de-epidermalised dermis (DED) is processed from cadaveric skin by removal of the epidermis and cellular components leaving behind essential extracellular matrix (ECM) and basement membrane proteins. DED is durable and has reduced or no antigenicity and the structural properties are retained even after cryopreservation [5], glycerol preservation [6] and lyophilisation [7]. Successful uses of DED for the resurfacing of full-thickness wounds have been described in detail [8]. DED has also been used in *in vitro* skin models with the incorporation of fibroblasts and keratinocytes [9, 10].

Integra^®^ Artificial Skin DE (C-GAG), used in the treatment of full-thickness burns [11, 12], comprises a bilayered porous dermal component of bovine collagen cross-linked with chondroitin-6-sulphate, attached to a silicone membrane which functions as a temporary epidermis [11, 12]. C-GAG requires a two-stage grafting procedure. Once applied to a freshly excised wound bed, completion of dermal regeneration takes up to 4 weeks, after which, the silicone layer is replaced by a thin split-skin graft to achieve permanent epidermal wound closure [13]. Cultured autologous keratinocytes [14], and cultured skin substitutes [15] have been used as second-stage procedures with this C-GAG in full-thickness wounds in humans.

Tissue engineered skin grafted onto full-thickness wounds provide complete wound closure however, hair follicles do not regenerate. In partial-thickness wounds, where remnants of skin appendages remain, wound closure can be achieved without the need for engraftment as the epidermis is regenerated from the hair stem cells that reside in the hair follicle bulge region [16].

We have previously investigated the clinical outcome of early implantation of whole hair follicles into a reconstructed C-CAG in the scalp region and showed that this effectively converted a full-thickness wound into a partial-thickness wound by creating a neo-dermis and restoring viable hair follicles. One year after transplantation, the neo-epidermis showed normal keratin 1 (K1) and K10 expression and the lack of epithelial hyperproliferation markers K6 and K16 [17].

In the present study we established *in vitro* skin models containing implanted hair follicles based on C-GAG or DED seeded with hair follicle-derived dermal papilla cells and investigated epithelial regeneration from outer root sheath (ORS) keratinocytes as well as follicular differentiation in comparison with those obtained *in vivo* from C-GAG implanted with whole hair follicles [17].

## Materials and methods

### Preparation of Integra^®^ artificial skin (C-GAG) and de-epidermalized dermis (DED)

Integra^®^ Artificial Skin DE (C-GAG) was commercially available from Integra^®^ (LifeSciences Corporation, Plainsboro, NJ) and DED was prepared from glycerol preserved skin (Euro Skin Bank, Beverwijk, The Netherlands). C-GAG and DED were prepared as described previously [18].

### Isolation and cell culture of dermal papilla cells

Informed written consent was obtained from all individuals who donated skin biopsies and collaborating dermatologists performed the biopsies at the Royal London Hospital (London, U.K.). The East London and City Health Authority Research Ethics Committee approved the use and protocols for obtaining patient skin biopsies (T/01/034). No minors were used for this study.

Cultured dermal papilla (DP) cells were included in all skin equivalents. DP cells were isolated from hair follicles from human scalp skin as previously described [19]. Two to four passage DP cells were used for experiments.

### Microdissection of human hair follicles

Human hair follicles from redundant scalp skin were isolated as either whole pilosebaceous units (PSU) comprising the hair follicle bulb, bulge region and sebaceous gland or, as truncated hair follicles (THF) consisting of the lower, proximal portion of the hair follicle excluding the bulge region and sebaceous gland as previously described [20, 21].

### Production of C-GAG and DED skin equivalents

1.5 × 1.5 cm squares of C-GAG and DED were placed in 12-well-plates, with the silicone surface (GAG) and the papillary dermal surface (DED) located at the bottom of the multiwell plate. 1 cm stainless-steel rings were placed on the DEs into which DP cells were resuspended in DP medium (2 × 10^5^ per cm^2^ in 500 ml). Medium was added to top up the rings and to the surrounding areas of the wells. After overnight incubation, the rings were removed and medium refreshed and DEs were incubated for a further 4 days. Following incubation, medium was removed and the DEs turned so that the silicone surface of the C-GAG and the papillary surface of the DED were now uppermost. Small slits were created in the DEs into which either 6 PSU or THF were inserted vertically into each DE. DEs were prepared in duplicates and experiments repeated three times (n = 6 per DE). The DEs were raised to air-liquid interface by draping the skin equivalents over stainless-steel rings placed on top of stainless-steel grids, allowing the hair follicles to remain in an upright position. The skin equivalents remained at air-liquid interface for 14 days with medium refreshed every 3 days (see S1 Fig). The skin equivalents were then cut into half embedded in 3% agar and fixed in 10% formalin and embedding in paraffin. Haematoxylin and eosin (H&E) histology and immunohistochemistry were performed according to standard laboratory procedures [18]. The primary antibodies used and their dilutions are shown in Table 1.

**Table 1:** Primary Antibodies used for Immunohistochemistry.

Antibody	Target	Dilution	Source
LHK1 (mouse monoclonal)	Keratin 1 (K1)	Supernatant	The Royal London School of Medicine & Dentistry [22]
LHK6 (mouse monoclonal)	Keratin 6 (K6)	Supernatant	The Royal London School of Medicine & Dentistry [22]
LHP2 (mouse monoclonal)	Keratin 10 (K10)	Supernatant	The Royal London School of Medicine & Dentistry [23]
LL002 (mouse monoclonal)	Keratin 14 (K14)	Supernatant	The Royal London School of Medicine & Dentistry [24]
LL025 (mouse monoclonal)	Keratin 16 (K16)	Supernatant	The Royal London School of Medicine & Dentistry [25]
Anti-Human β-catenin–Pan (C-14) (mouse monoclonal)	Pan β-catenin	1:100	BD BioScience product no. 610153
Anti-human PCNA (PC10) (mouse monoclonal)	PCNA	1:100	DAKO product no. M 0879
Anti-human p63 (mouse monoclonal)	p63	1:100	Santa Cruz Biotechnology, Inc. product no. sc-8431
Anti-human collagen type-IV (rabbit polyclonal)	Collagen IV	1:50	ICN Pharmaceuticals, Inc., product no. 10760
Anti-human vimentin (V9) (mouse monoclonal)	Vimentin	1:100	DAKO product no. M 0725

### Micrografting hair follicles into the engrafted C-GAG

The two-step procedure of engrafting the C-GAG onto the burn wound followed by micrografting hair follicles into the engrafted C-GAG has previously been described [17]. Briefly, autologous PSU were dissected from a section of the occipital scalp and micrografted through the silicone sheet of C-GAG at intervals of 5 to 10 mm. Biopsy specimens were taken at 16 days, 37 days, 1 and 2 years post PSU micrografting fixed in 10% formalin and processed for histology. The biopsy specimens had been obtained from our previously published study [17]. Normal scalp skin from a donor was used as control scalp skin with approval from the East London and City Health Authority Research Ethics Committee as well as informed written consent from the patient as previously mentioned.

## Results

### Histology of engrafted C-GAG *in vivo*

**Fig 1:**
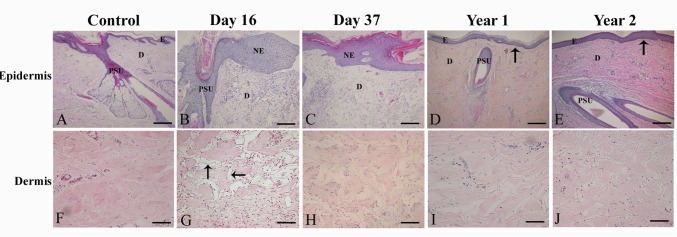
**H&E histology of control scalp skin (A, F), biopsies at day 16 (B, G), day 37 (C, H), 1 year (D, I) and 2 years (E, J) post PSU micrografting.** The general histology of control scalp skin and biopsies taken at different time-points are shown in the top panels (A-E). The bottom panels show the histology of the dermis of control scalp skin and biopsies taken at different time-points (F-J). C-GAG fibres are present in the dermis of all biopsies taken between day 16 (arrows) and 2 years post PSU micrografting (G-J). Scale bar (A-J), 200μm. Abbreviations: E, epidermis; D, dermis, NE, neo-epidermis; PSU, pilosebaceous unit. Arrows denote dermo-epidermal junctions. Fig 1B and 1C are reprinted from [17] under a CC BY license, with permission from [Wolters Kluwer Health, Inc.], original copyright [2004]. *Use of the material in print, digital or mobile device format is prohibited without the permission from the publisher Wolters Kluwer. Please contact healthpermissions@wolterskluwer.com for further information.*

We previously investigated the clinical outcome of early implantation of autologous PSU into a vascularised C-GAG neo-dermis on a burns patient and examined the general histology of the grafted site 16 and 37 days post grafting [17]. We now expanded this analysis and investigated both histology of the grafted site in biopsies taken 1 and 2 years after grafting in comparison to control scalp skin (Fig 1A and 1F).

At 16 days post micrografting, neo-epithelium originating from implanted hair was visible (Fig 1B, and previously published [17]). A well vascularised neo-dermis was apparent with evidence of C-GAG collagen fibres still present (Fig 1G -arrows). Biopsy taken at 37 days post micrografting showed complete re-epithelialisation with a neo-epidermis 8 to 9 cell layers thick (Fig 1C, and previously published [17]). The neo-dermis contained remnants of collagen fibres of the C-GAG matrix (Fig 1H). Biopsies taken at 1 and 2 years post micrografting exhibited a similar morphology and architecture to that of control scalp skin (Fig 1D, 1E, 1I and 1J). At 1 and 2 years post micrografting, a relatively flat dermo-epidermal junction was seen (Fig 1D and 1E; arrows) and few collagen fibres of the C-GAG matrix were still present (Fig 1I and 1J).

### Histology of skin equivalents derived from PSU and THF

**Fig 2:**
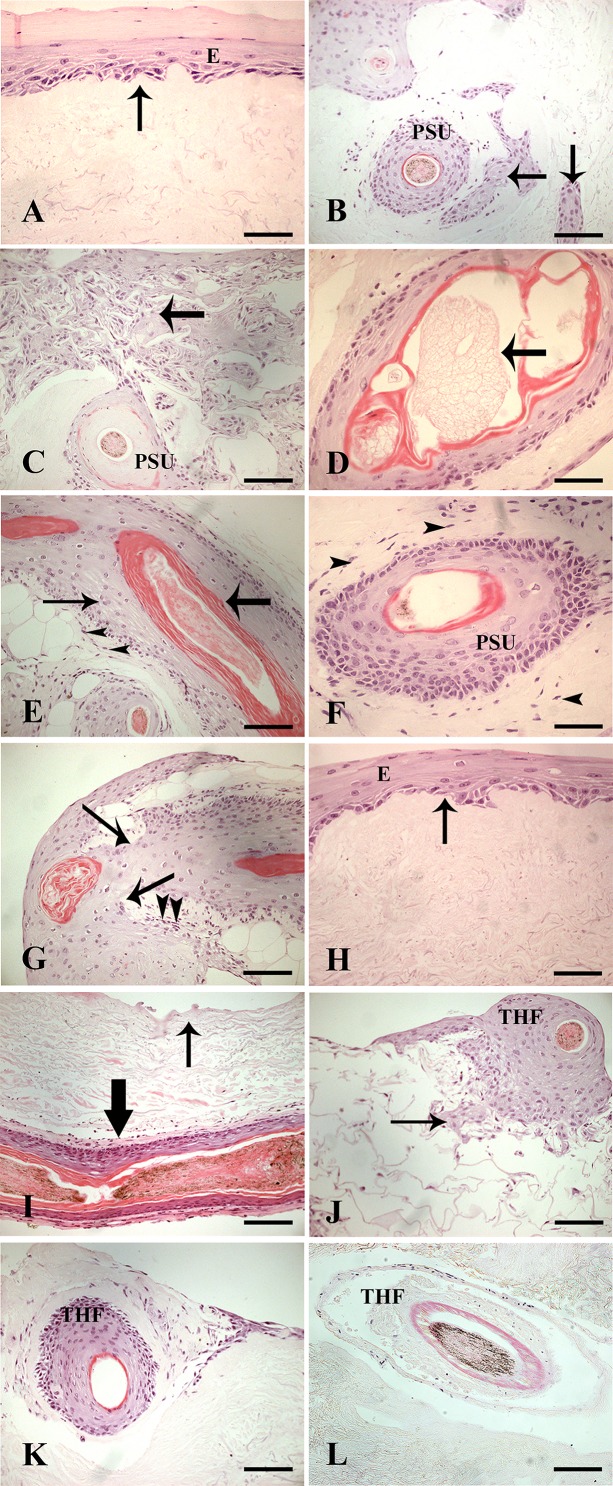
**H&E histology of skin equivalents implanted with PSU (A-G) or THF (H-L).** Epidermis originating from ORS cells from implanted PSU in DED (A). Islands of cells present inside DED (B; arrows). Large cluster of cells present in C-GAG matrix (C; arrow). Abnormal sebaceous gland (D; arrow). Lower region of PSU showing thick IRS (thick arrow), thick ORS (thin arrow) and intact CTS (arrowheads) (E). Cross-section of implanted PSU showing maintained morphology and thicker CTS (F; arrowheads). ORS cell outgrowth from PSU (arrows) (G). Epidermis present in DED implanted with THF (H). DED showing a lack of epidermis on papillary DED (thin arrow) despite presence of hair follicle (thick arrow) (I). Smaller clusters of cells present in the C-GAG matrix (J; arrow). Cross-section of healthy THF after implantation into DED (K). Cross-section of unhealthy THF after implantation into DED (L). Scale bar for (A), (D), (F) and (H), 50 μm; (B), (C), (E), (G) and (I-L), 100 μm. Abbreviations: E, epidermis; PSU, pilosebaceous unit, THF, truncated hair follicle. Arrows denote dermo-epidermal junctions.

DED implanted with PSU after 14 days at the air-liquid interface showed a multilayered epidermis on the papillary surface. The dermo-epidermal junction was relatively flat (Fig 2A; arrow). Most DED also contained islands of cells within the dermis (Fig 2B -arrows). C-GAG implanted with PSU did not generate a multilayered epidermis, instead, cells migrating from the PSU formed islands within the matrix (Fig 2C). The sebaceous glands showed abnormal differentiation including cell nuclei loss and a keratinised lumen (Fig 2D). PSU generally had a thicker inner (IRS) and outer root sheath (ORS) (Fig 2E -arrows). Generally, PSU morphology was maintained at proximal (lower) regions as identified by the absence of ORS explant and presence of an intact connective tissue sheath (CTS) (Fig 2E -arrowheads) however, the latter appeared thicker (Fig 2F). However, at the distal (upper) ends where there was sometimes a small amount of epidermis attached to PSU, keratinocyte outgrowth was clearly seen (Fig 2G–arrows).

H&E histology of DED implanted with THF was variable. Out of a total of 36 THF implanted into 6 DEDs, 29 THF explanted to generate a multilayered epidermis on DED (Fig 2H) whereas 7 THF did not show any signs of epidermal regeneration despite their presence in DED (Fig 2I). C-GAG implanted with THF did not generate an epidermis (Fig 2J).

Fewer cell islands were observed in the interstices of C-GAG implanted with THF compared to those implanted with PSU (Fig 2J versus Fig 2C). Histology of THF inside DED and C-GAG was variable. In DED, the majority of THF exhibited a similar morphology to that described for PSU (Fig 2K). However, a few THF appeared to have undergone abnormal differentiation resulting in cell death as demonstrated by loss of cell nuclei (Fig 2L). These were not investigated further. In C-GAG scaffold, THF analysed showed an abnormal morphology as a result of cells explanting from the ORS but did not form cell islands (Fig 2J).

### Hyperproliferation and differentiation profile of skin equivalents and neo-epidermis *in vivo*

**Fig 3:**
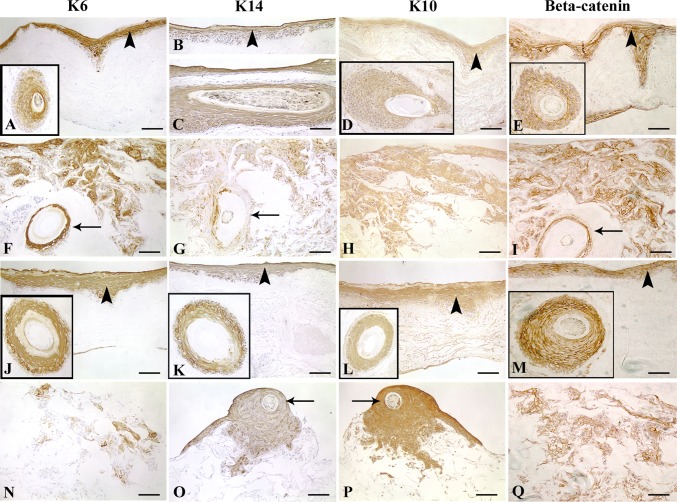
**Expression of K6 (A, F, J, N), K14 (B, C, G, K, O), K10 (D, H, L, P) and** β**-catenin (E, I, M, Q) in DEs implanted with PSU (A-I) or THF (J-Q).** K6 expression seen in suprabasal epidermal layers in DED implanted with PSU (A) and THF (J). PSU in DED exhibit suprabasal ORS staining (A -inset). Similar staining seen for THF in DED (J -inset). Groups of positive K6 ORS cells found in C-GAG matrix implanted with PSU (F). K6 positive PSU implanted into C-GAG matrix is denoted by arrow (F). Staining also observed with THF C-GAG skin equivalents (N). K14 staining found in epidermis of DED implanted with PSU (B) and THF (K). PSU exhibit basal and suprabasal ORS staining in DED (C). The same is true for THF in DED (K -inset). In C-GAG, islands of K14 positive ORS cells seen inside matrix implanted with PSU (G) or THF (O). These hair follicles are also K14 positive (G, O; denoted by arrows). K10 staining seen in epidermis of DED implanted with PSU (D) and THF (L). K10 staining seen in the suprabasal ORS of PSU (D -inset) and THF (L–inset) implanted into DED. K10 positive ORS cells found in C-GAG scaffold implanted with PSU (H) or THF (P). THF implanted into C-GAG matrix is denoted by arrow (P). Membranous β-catenin staining found in the epidermis of DED implanted with PSU (E) and THF (M). In DED, implanted PSU (E -inset) and THF (M -inset) exhibit membranous ORS staining. In C-GAG scaffold, islands of ORS cells show membranous staining inside the matrix implanted with PSU (I) or THF (Q). Implanted PSU are positive for β-catenin (I, arrow). Scale bar for (A-Q), 100 μm. Arrowheads denote epidermis.

K6 a marker for hyperproliferation and K14 a basal marker for stratified squamous epithelia are expressed in a similar fashion in *in vitro* skin models as they are in wound healing and hyperproliferative skin diseases [18, 26]. As expected, K6 and K14 expression was found in both DED and C-GAG skin equivalents. Strong K6 expression was mainly in the suprabasal layers of the regenerative epithelium in DED implanted with PSU and THF similar to wound healing skin (Fig 3A and 3J). Both PSU and THF expressed K6 in the suprabasal ORS (Fig 3A and 3J -insets). PSU implanted into C-GAG displayed K6 staining in the inner C-GAG matrix and in the suprabasal ORS (Fig 3F). Clusters of K6 positive cells were found in the C-GAG matrix implanted with THF (Fig 3N). These THF also expressed K6 (not shown). K14 was expressed throughout the epidermis of DED implanted with PSU and THF and in both basal and suprabasal layers of the ORS (Fig 3B, 3C and 3K- insert). In C-GAG, K14 positive ORS keratinocytes were observed explanting from PSU and THF (Fig 3G and 3O) and in both PSU and THF (Fig 3G and 3O).

**Fig 4:**
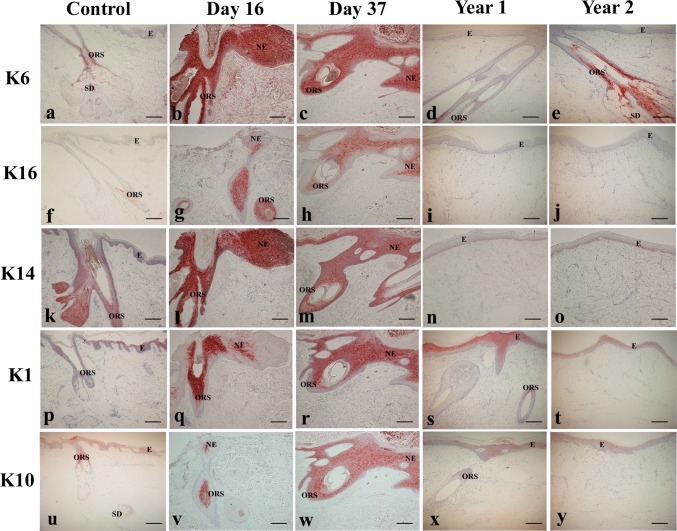
**Hyperproliferation (K6 and K16), basal epidermal (K14) and differentiation (K1 and K10) profiles in control scalp skin (A, F, K, P, U), biopsies taken at day 16 (B, G, L, Q, V), day 37 (C, H, M, R, W), 1 year (D, I, N, S, X) and 2 years (E, J, O, T, Y) post PSU micrografting.** Immunohistochemistry was performed using antibodies directed against human K6, K16, K14, K1 and K10 as seen in Table 1. Expression of these markers was found to be similar to control skin by 1 and 2 years post micrografting. Scale bar (A-Y), 200 μm. Abbreviations: E, epidermis; ORS, outer root sheath; SD, sebaceous duct; NE, neo-epidermis. Fig 4S and 4X are reprinted from [17] under a CC BY license, with permission from [Wolters Kluwer Health, Inc.], original copyright [2004]. *Use of the material in print, digital or mobile device format is prohibited without the permission from the publisher Wolters Kluwer. Please contact healthpermissions@wolterskluwer.com for further information.*

*In vivo*, K6 and K16 were not present in any of the layers of the epidermis in control scalp skin but were observed throughout the suprabasal layers of the ORS and sebaceous duct (K6) (Fig 4A and 4F). In contrast, at day 16 post micrografting, the suprabasal layers of the neo-epithelium including the leading edge and ORS stained strongly for K6 (Fig 4B) and weakly for K16 (Fig 4G). A similar pattern of distribution for K6 and K16 was seen at day 37 post micrografting (Fig 4C and 4H). The expression patterns for K6 and K16 at 1 and 2 years post micrografting were similar to those seen for control scalp skin (Fig 4D, 4E, 4I and 4J). K14 expression was restricted to the basal and 1 or 2 suprabasal layers in control scalp skin and in both basal and suprabasal layers of the ORS and in the sebaceous gland (Fig 4K). At days 16 and 37 post micrografting, K14 staining increased in intensity and also extended throughout all the suprabasal layers of the neo-epidermis. Expression was also found in the basal and suprabasal layers of the ORS (Fig 4L and 4M). Staining for K14 at 1 and 2 years post micrografting resembled that of control scalp skin (Fig 4N and 4O).

K10 a marker of differentiation was detected in the suprabasal layers of the epidermis in DED implanted with PSU (Fig 3D) and similar expression profile was observed in DED implanted with THF (Fig 3L). PSU and THF within the DED expressed K10 in the suprabasal layers of ORS (Fig 3D -inset and 3L -insert). In C-GAG, K10 was expressed in clusters of ORS cells that had migrated from PSU or THF (Fig 3H and 3P). K10 localisation in PSU implanted into C-GAG was similar to those found in DED (not shown). However, most THF implanted into C-GAG exhibited K10 staining throughout the ORS (Fig 3P).

In comparison, *in vivo*, K1 was moderately expressed and K10 was weakly expressed in the suprabasal layers of the epidermis in control scalp skin. Expression was also found in the upper part of the ORS just above the sebaceous glands (Fig 4P and 4U). At day 16 post micrografting, K1 and K10 were localised to the suprabasal layers of the neo-epithelium except at the leading edge where keratinocyte cells were negative suggesting that the cells were undifferentiated. Suprabasal cells of the ORS also stained positive for K1 and K10 (Fig 4Q and 4V). Similar expressions of both these keratins were found in all suprabasal layers indicating the presence of differentiated keratinocytes at day 37 post micrografting (Fig 4R and 4W). As previously published for 1 year post micrografting [44], K1 and K10 expression was identical to control scalp skin indicating a return to the normal differentiation state (Fig 4S and 4X). Results obtained for 2 years post micrografting were similar (Fig 4T and 4Y).

### Presence of adherens junctions in skin equivalents

Membranous β-catenin was seen along intercellular junctions of the epidermis, reducing in intensity in the basal layers of DED implanted with PSU and THF (Fig 3E and 3M). Implanted PSU and THF (Fig 3E–inset and 3M -inset) also showed membranous staining in the ORS. Keratinocyte clusters within the C-GAG matrix implanted with PSU and THF (Fig 3I and 3Q) exhibited membranous staining. Staining for β-catenin in these hair follicles was similar to those implanted in DED.

### Keratinocyte proliferation in the skin equivalents and *in vivo*

**Fig 5:**
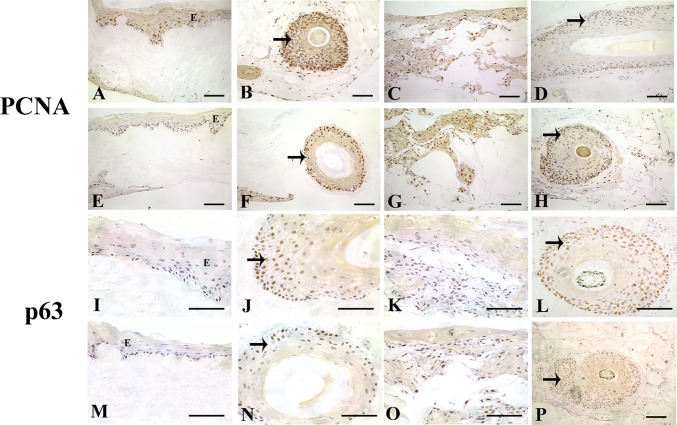
**PCNA (A-H) and p63 (I-P) staining in DED and C-GAG skin equivalents implanted with PSU (A-D, I-L) or THF (E-H, M-P).** PCNA staining seen in the epidermis of DED implanted with PSU (A) and THF (E). PSU (B) and THF (F) implanted into DED are also positive for PCNA. C-GAG skin equivalents derived from PSU (C) and THF (G) exhibit PCNA staining. PSU (D) and THF (H) implanted into C-GAG contain PCNA positive cells. p63 staining seen in the epidermis of DED implanted with PSU (I) and THF (M). PSU (J) and THF (N) implanted into DED are positive for p63. C-GAG skin equivalents derived from PSU (K) and THF (O) display p63 staining. PSU (L) and THF (P) implanted into C-GAG contain p63 positive cells. Scale bar for (A-P), 100 μm. Abbreviations: E, epidermis. Arrows denote ORS of hair follicles.

To investigate keratinocyte proliferation in the skin equivalents, proliferating cell nuclear antigen (PCNA) and p63 expression was assessed. p63 was also used as a putative stem cell marker to determine the presence of epithelial progenitor cells. PCNA and p63 staining were found in nuclei of cells predominantly in the basal layer of the epidermis in DED implanted with PSU (Fig 5A and 5I) and THF (Fig 5E and 5M). Both hair follicle types expressed PCNA (Fig 5B and 5F) and p63 in the ORS (Fig 5J and 5N). C-GAG displayed PCNA and p63 staining for some keratinocytes situated in clusters inside the matrix when implanted with PSU (Fig 5C and 5K) and THF (Fig 5G and 5O). Both types of hair follicles implanted into the C-GAG matrix expressed PCNA (Fig 5D and 5H) and p63 (Fig 5L and 5P).

**Fig 6:**
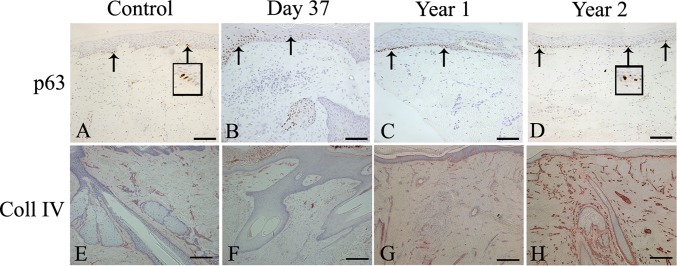
**Expression of p63 and Collagen type-IV in control scalp skin (A, E), biopsies taken at day 37 (B, F), 1 year (C, G), and 2 years (D, H) post PSU micrografting.** Immunohistochemistry was performed using antibodies directed against human p63 and collagen type-IV as seen in Table 1. At 1 and 2 years post PSU micrografting, p63 and collagen type-IV expression were similar to control skin. Scale bar (A-H), 200 μm. Arrows showing p63 positive cells in the basal layer of the epidermis.

In control scalp skin, p63 expression was seen in the basal layer of the epidermis (Fig 6A). Expression of p63 at day 37 was found mainly in the basal layer (Fig 6B). By 1 and 2 years post micrografting, staining was again restricted mainly to the basal layers of the epidermis like that of control scalp skin (Fig 6C and 6D). No further sections could be obtained for day 16 sample as all clinical material had been used up.

### Expression of ECM component and mesenchymal marker in skin equivalents and *in vivo*

**Fig 7:**
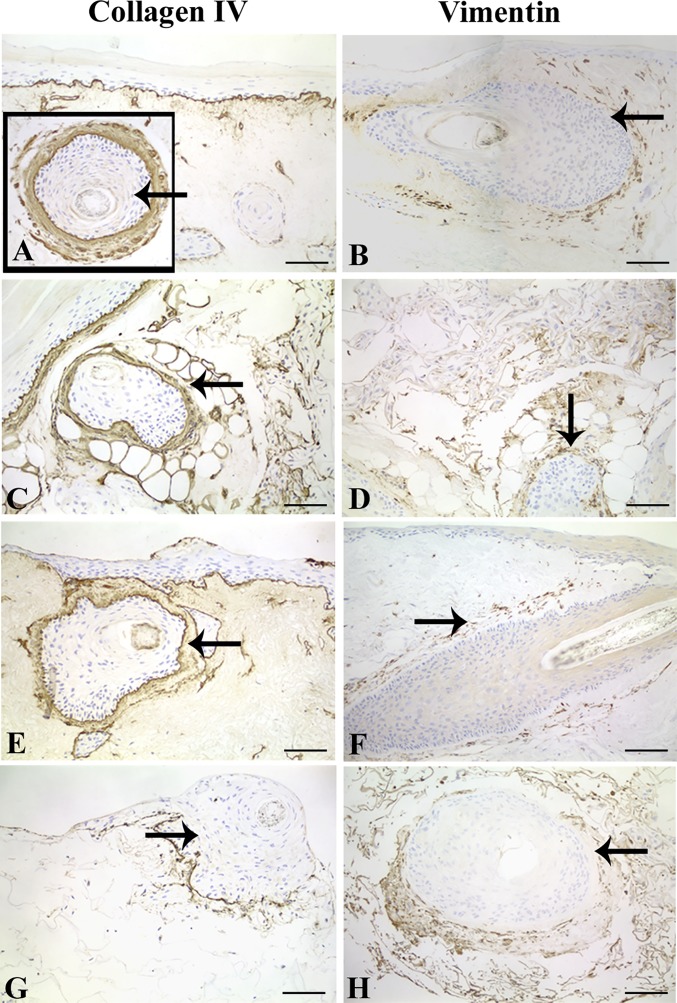
**Collagen type-IV and vimentin staining in DED and C-GAG skin equivalents implanted with PSU (A-D) or THF (E-H).** Collagen type-IV staining was found at basement membrane zones in DED implanted with PSU (A) and around PSU (A -inset). This was also true for DED skin equivalents implanted with THF (E). C-GAG matrix implanted with PSU show positive staining at basement membranes around hair follicle and amongst cell clusters in the matrix (C). C-GAG matrix implanted with THF show positive staining along basement membrane zones of hair follicles and in association with some cells inside the matrix (G). In DED, vimentin positive cells were found in the CTS of implanted PSU (B) and THF (F). Positive cells were also seen within DED, away from hair follicles (B, F). Vimentin positive cells were observed within C-GAG matrix and around implanted PSU (D) and THF (H). Arrows denote implanted PSU and THF. Scale bar for (A-H), 100 μm.

Collagen type-IV stained the dermo-epidermal junction and areas previously colonised by blood vessels in DED implanted with PSU (Fig 7A) and THF (Fig 7E). Collagen type-IV also stained the basement membranes surrounding PSU (Fig 7A–inset) and THF (Fig 7E). Dense collagen type-IV staining was also seen in association with the CTS (Fig 7A–inset and 7E -arrow). Collagen type-IV was found in association with some ORS cell clusters within the C-GAG matrix implanted with THF and PSU as well as around hair follicle basement membranes (Fig 7C and 7G).

I*n vivo*, collagen-type-IV was found in conjunction with the basement membrane along the dermo-epidermal junction, the ORS, the sebaceous glands and the sweat glands in control scalp skin and blood vessels (Fig 6E). At day 37 post micrografting, Collagen type-IV was observed in association with blood vessels. No expression was observed at the dermo-epidermal junction of the neo-epidermis (Fig 6F). At 1 and 2 years post micrografting, Collagen type-IV was distributed in a similar manner to that of control scalp skin (Fig 6G and 6H).

Strong vimentin staining was found in association with CTS surrounding both PSU and THF implanted into DED (Fig 7B and 7F). Vimentin positive cells were also found inside the DED, presumably seeded DP cells and explanted CTS cells (Fig 7B and 7F). C-GAG implanted with PSU and THF showed positive staining of CTS surrounding the hair follicles and vimentin positive cells within the matrix (Fig 7D and 7H).

## Discussion

In this paper, we have established *in vitro* organotypical skin models containing hair follicles based on DED or C-GAG DEs to mimic the natural topography of hair bearing skin [27, 28].

PSU implanted into DED gave rise to a multilayered epidermis that expressed markers similar to mature epidermis and those of wound healing skin as confirmed by K6, K10, K14 and β-catenin staining [29, 30]. The epidermis was separated from the dermis by the basement membrane, confirmed by collagen type-IV expression at this location, which assisted in the attachment, proliferation and differentiation of the keratinocytes confirming other studies [10, 18]. Islands of ORS cells were frequently found inside the DED skin equivalents probably due to ORS keratinocytes invading the spaces previously colonised by the appendageal structures inherent to DED.

In contrast, the formation of an organised epidermis with C-GAG was not observed. This may be attributed to the lack of a proper basement membrane zone and the presence of large pores of the C-GAG matrix permitting the infiltration and proliferation of cells, one of its main functions *in vivo* [31]. Thus, arrangement of ORS keratinocytes in the C-GAG matrix was disorganised and although expression of differentiation and hyperproliferation markers was observed, their localisation was abnormal. The failure of C-GAG to support epidermal regeneration *in vitro* suggests *in vivo* factors are essential for full epidermal regeneration using C-GAG. In support of this, we previously reported that implanting hair follicle micrografts into the same C-GAG neo-dermis on a full-thickness scalp burn wound gave rise to a normal multilayered, differentiated epidermis which was derived from ORS cells [17]. At the early time points days 16 and 37 post micrografting, K6, K10, K14 and p63 expression was very similar to those obtained with DED implanted with PSU confirming that this is a useful *in vitro* model to study wound healing skin. The novel technique of implanting PSU into vascularised C-GAG effectively converted a full-thickness wound to deep partial-thickness thus obviating the need for split-skin grafts. These results are in alignment with previous studies that have also shown epidermal regeneration from appendages in partial-thickness wounds [32, 33].

29 out of 36 THF implanted into DED led to the formation of an epidermis. The reason for the lack of epithelial regeneration in some of the THF implants may be due to extensive cell death in some hair follicles, demonstrated by absence of ORS cell nuclei. It was also noted that some ‘healthy’ THF moved underneath the papillary surface of the DED during culture making it difficult for the ORS cells to migrate onto the papillary surface. In this case, islands of cells were seen in the slits created in the DED where hair follicles were inserted. THF implanted into C-GAG failed to form an epidermis.

Histology revealed that the integrity of the IRS, ORS, CTS and basement membrane compartments of the implanted PSU appeared to be maintained although some morphological changes were observed at regions proximal to the infundibulum including thicker CTS, ORS and IRS. Thicker IRS has also been observed in hair follicles that have been in culture for 9 days [34]. At the distal end of the hair follicles however, the hair follicle structure, in particular the ORS compartment, was not maintained. ORS cell explantation was expected due to the presence of serum in the culture medium which promotes cell attachment, proliferation and differentiation. In DED, ORS cells migrated laterally along the papillary surface of the dermis only invading the dermis at openings of old appendageal structures. Indeed, other studies have shown the upwards and lateral migration of ORS cells to regenerate the epidermis in a murine wound repair model [35], in a chimeric vibrissal follicle-transplantation study [36], and in partial-thickness burn wounds in humans [37]. With C-GAG, it is likely that these cells migrated upwards and out of the infundibulum and then back down inside the matrix due to the lack of basement membrane zone.

Further confirmation that the ORS compartments and basement membrane zones were maintained in PSU, at least in the lower regions, was achieved by immunostaining for K6, K10, K14, β-catenin and collagen type-IV that are normally expressed in the hair follicles. The expression patterns of these markers in these hair follicles were similar to their counterparts *in vivo*. These results are in keeping with another study that reported maintenance of hair follicle structure using their skin equivalent system comprising of PSU implanted into fibroblast sheets seeded with keratinocytes [38].

C-GAG implanted with PSU generally contained more ORS cells within the matrix compared to those implanted with THF. This may be due to PSU being larger in size compared to THF or the fact that more ORS keratinocytes were generated from PSU compared to THF could be an indication that the highly proliferative cells originated from the upper part of the hair follicle. Our results are consistent with clonal assays that have demonstrated that cells with higher proliferative potential, and prolonged viability are found in the upper region including the bulge of human hair follicles compared to lower regions [36, 39]. Most THF also gave rise to proliferative ORS cells that were able to generate an epidermis on DED. This is in line with previous work [40]. On the whole, our study demonstrated that PSU are the follicles of choice to use in these skin models as they are whole hair follicles and contain cells of all varying proliferative capacities both at the upper and lower ends of the hair follicle.

The importance of the dermal papilla and cultured DP cells in the induction of hair follicle activity and maintenance is well established [41, 42]. For this reason, hair follicles containing intact bulbs and cultured DP cells were also included in our system. Positive vimentin staining seen throughout the DED and C-GAG matrix indicated the presence of mesenchymal cells presumably, seeded DP cells and explanted CTS cells originating from the hair follicles. Other studies indeed have demonstrated fibroblast outgrowth from dermal sheath of hair follicles in the presence of serum [43]. As expected therefore, we observed increased collagen type-IV staining found in association with mesenchymal cells in our study. Very little information on hair follicle bulb morphology was obtained in this study because of the difficulty in obtaining longitudinal sections of the implanted hair follicles, however it was not the aim of this study to investigate hair growth as we included FCS in our culture medium.

## Conclusions

Skin models are useful tools to study various aspects of skin biology, physiology, renewal, wound repair and regeneration and skin diseases. However, it should be noted that they do not mimic every element of native skin nor do they replace all of its functions. These models are deprived of essential communication with the rest of the body including neuroendocrine communications that play an integral role as central regulators of body homeostasis [44, 45]. They also lack functional immune system and regulation and, blood flow important for cellular nutrition and metabolism [46]. The development of an ideal, fully functional skin model has therefore not been attained yet. However, over the years, ongoing research into the cellular and molecular mechanisms which govern the various processes during skin development, repair, renewal and regeneration has led to major advancements in the construction of these skin models that more closely resemble native skin by the incorporation of a variety of cell types including neuronal cells [47], immune cells, melanocytes, endothelial cells, and hair follicle-like structures [48, 49].

In this study, we have established an *in vitro* skin model that mimics the conditions *in vivo* to study epithelial/mesenchymal interactions and epithelial regeneration from hair follicles. Both DEs supported the initial maintenance of implanted hair follicles, in particular PSU. These follicles provided progenitor epithelial cells that generated epidermis only in DED skin equivalents similar to *in vivo*. The skin equivalents implanted with PSU were our model of choice. A superior morphology was associated with DED skin equivalent implanted with PSU *in vitro*. Based on our *in vitro* and *in vivo* studies, we conclude that micrografting of PSU into tissue engineered skin may highlight an alternative method to skin grafting of full-thickness wounds. This could be the way forward for the treatment of wounds, potentially saving time and split-skin donor sites.

## Supporting information

S1 Fig Production of C-GAG and DED Skin Equivalents. (JPG)
