# Supplementary figures and images for: In vitro skin models to study epithelial regeneration from the hair follicle
Source: PLoS One. 2017 Mar 28;12(3):e0174389. doi: 10.1371/journal.pone.0174389 (PMC5370106; doi:10.1371/journal.pone.0174389)

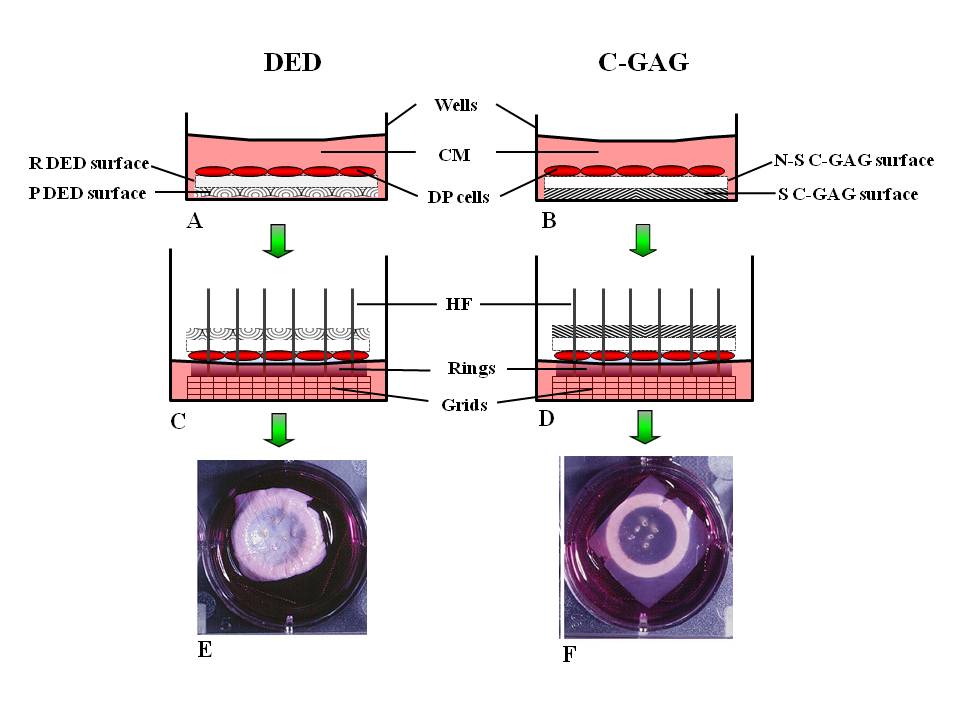

Supplement: S1 Fig — (JPG) [file pone.0174389.s001.jpg]
